# Combined Use of the Hemi-Split Tibialis Anterior Muscle and Soleus Muscle Flap for Reconstruction After the Excision of a Recurrent Soft Tissue Sarcoma in the Lower Leg

**DOI:** 10.7759/cureus.69145

**Published:** 2024-09-11

**Authors:** Daisuke Atomura, Takeo Osaki, Shunsuke Sakakibara

**Affiliations:** 1 Plastic Surgery, Kobe University Hospital International Clinical Cancer Research Center, Kobe, JPN; 2 Plastic Surgery, Hyogo Cancer Center, Akashi, JPN; 3 Plastic Surgery, Kobe University Graduate School of Medicine, Kobe, JPN

**Keywords:** lower leg reconstruction, pedicled muscle flap, soft tissue sarcoma, soleus muscle flap, tibialis anterior muscle flap

## Abstract

Reconstruction of large tissue defects following soft tissue sarcoma (STS) resection in the lower extremity presents a significant challenge for plastic surgeons. The optimal method should be selected from a wide array of techniques, depending on the size and location of the defect. Both pedicled and free flaps have demonstrated favorable outcomes. However, due to the nature of STS, there is concern about recurrence and the potential complications for secondary reconstruction. A 78-year-old male with leiomyosarcoma in the proximal third of the leg underwent wide excision and reconstruction using a pedicled medial gastrocnemius muscle flap. Local recurrence occurred at six months postoperatively, necessitating a second extended resection. This was followed by reconstruction using a hemi-split tibialis anterior muscle and hemi-split soleus muscle flaps, combined with a split-thickness skin graft. The postoperative course was uneventful, with complete survival of both the muscle flap and skin graft and no functional limitations in the lower extremity. The hemi-split tibialis anterior and soleus muscle flaps are considered valuable methods for reconstruction, preserving lower extremity function.

## Introduction

Reconstruction of extensive tissue defects following soft tissue sarcoma (STS) resection in the lower extremity presents a significant challenge for plastic surgeons [1]. The lack of available surrounding tissue complicates the search for an appropriate reconstructive solution. In the anterior part of the lower leg, particularly, the limited subcutaneous tissue and immediate exposure of bone and tendons posttumor resection necessitate flap reconstruction [2,3]. Pedicled flaps are selected based on defect location: the proximal third is typically reconstructed using a local gastrocnemius muscle flap with a skin graft, the middle third with soleus muscle flaps, and the distal third with a sural flap. The gastrocnemius flap offers versatility when both muscle and skin coverage are required for the proximal third of the leg and around the knee [4]. Its dissection and harvest are rapid, are technically accessible to reconstructive surgeons without necessitating microsurgical vascular anastomosis, and yield favorable functional outcomes [5,6].

Local recurrence of STS remains a significant concern, with reported rates ranging from approximately 6.5% to 25% [7]. Fujiki et al. reported a recurrence rate of 14.4% following STS reconstruction [8]. Complete surgical resection with microscopically clear margins is desirable in patients with locally recurrent STS [9]. Although treatment of recurrent cases typically involves re-excision and reconstruction, the second surgery is more challenging than the initial procedure due to several factors: previously used reconstructive tissue is unavailable; vessels suitable for pedicled or free flaps have been depleted; and tissue has been scarred by prior surgery or postoperative radiotherapy. Furthermore, radical resection and reconstruction using the remaining tissue may result in substantial functional impairment of the extremity.

Here, we report a case of lower leg leiomyosarcoma that recurred after reconstruction with a gastrocnemius muscle flap. Subsequent reconstruction was performed using a hemi-split tibialis anterior muscle flap and soleus muscle flap combined with a skin graft, resulting in favorable functional outcomes.

## Case presentation

**Figure 1:**
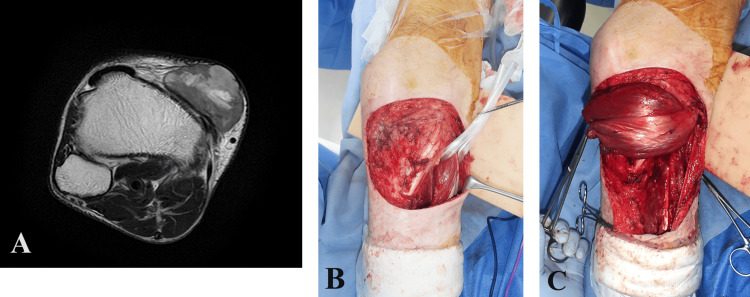
(A) Magnetic resonance imaging revealed a mass located in the proximal anterior compartment of the leg. (B) Following tumor excision, a portion of the tibia and tendon were exposed. (C) The medial gastrocnemius muscle pedicled flap covered the bone and tendon.

A 78-year-old male patient with a history of simple resection of leiomyosarcoma in the proximal third of the right lower leg was referred to our hospital. Preoperative magnetic resonance imaging revealed a 3.8 cm mass located in the anterior aspect of the proximal leg (Figure 1A). Orthopedic surgeons performed a wide resection of the tumor with a 2 cm margin from the tumor edge and above the tibial periosteum. The defect was reconstructed using a pedicled medial gastrocnemius muscle flap with a split-thickness skin graft (Figure 1B, 1C).

**Figure 2:**
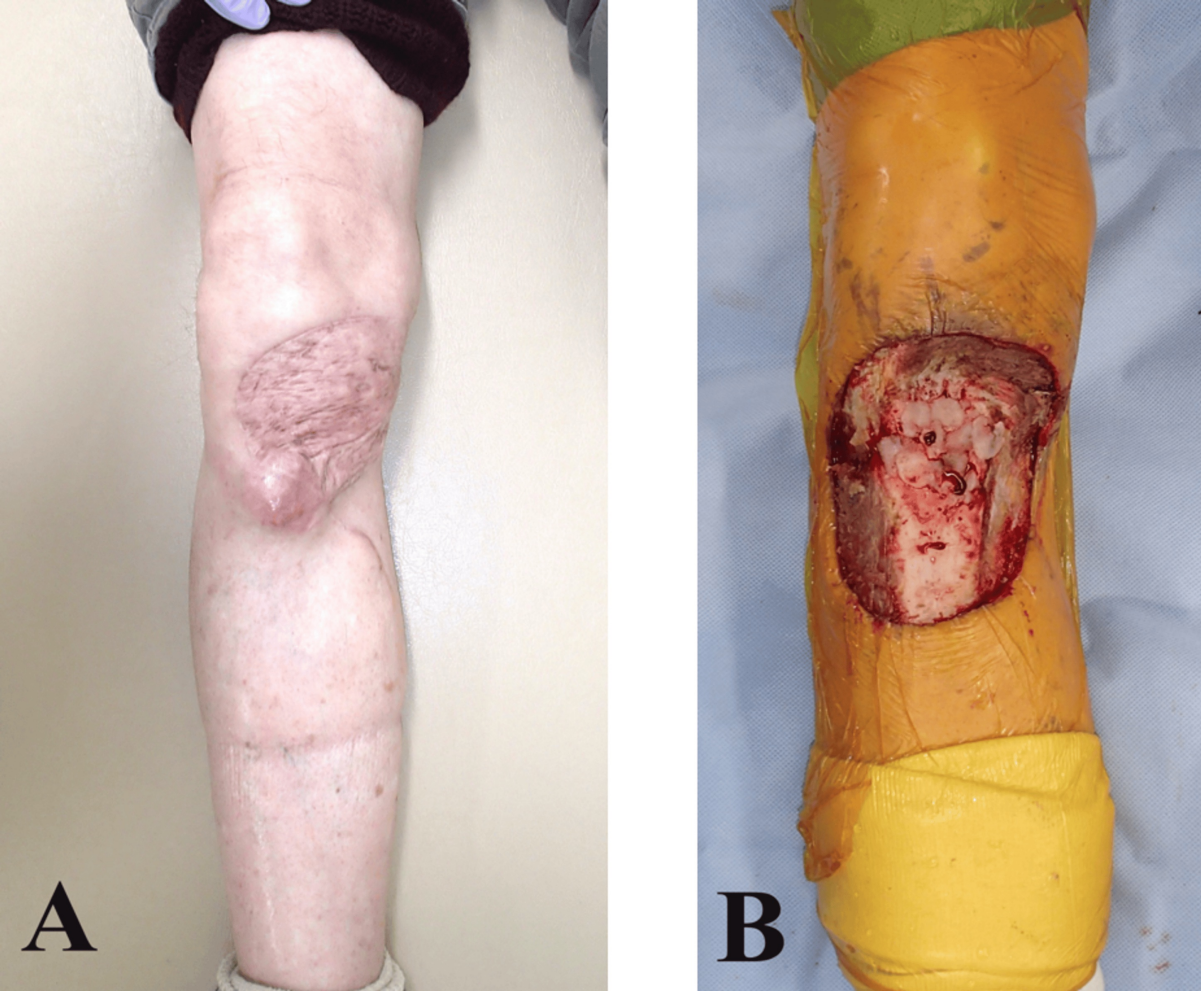
(A) Six months postsurgery, an enlarged lesion was detected at the margin of the reconstructed area, indicating local recurrence. (B) Following tumor resection, a skin defect measuring approximately 11 cm in diameter was observed, exposing the proximal tibia and part of the patellar tendon.

**Figure 3:**
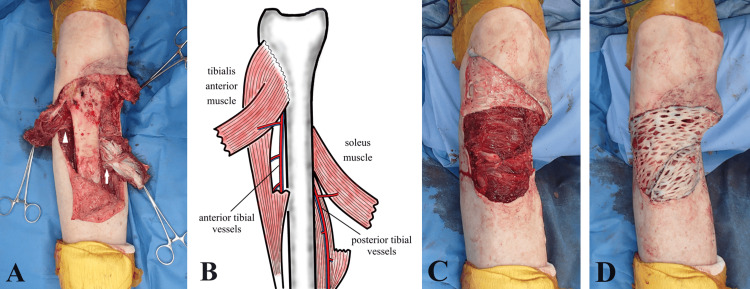
(A) The hemi-split tibialis anterior muscle flap and soleus muscle flap were elevated, supplied by branches of the anterior tibial vessels (white arrowhead) and posterior tibial vessels (white arrow), respectively. (B) Schematic drawing showing the hemi-split tibialis anterior muscle and soleus muscle flap with each dominant vessel. (C) The elevated tibialis anterior muscle and soleus muscle flap were sutured to cover the tibia. (D) A split-thickness skin graft was applied over them. Image Credits: Daisuke Atomura

Six months postoperatively, an enlarged lesion was detected at the edge of the reconstructed area, raising suspicion of local recurrence (Figure 2A). Extended resection was performed again. The tumor was resected en bloc with a 3 cm margin by orthopedic surgeons, including the medial gastrocnemius muscle flap used in the initial reconstruction. Proximally, the paratenon of the patellar tendon was also resected. After tumor resection, a skin defect measuring approximately 11 cm in diameter was observed, exposing the proximal tibia and part of the patellar tendon (Figure 2B). The defect was relatively large, and a single muscle flap was insufficient for coverage. The tibialis anterior muscle surroundings were dissected, and segmental branches from the anterior tibial vessels were identified. The amount of harvested muscle was adjusted by manually pinching the muscle belly and moving the ankle joint, deciding on approximately half the muscle width. Including the proximal vascular pedicle, the muscle body was cut distally, and the proximally based hemi-split tibialis anterior muscle flap was elevated. The soleus muscle surroundings were dissected, and among the branches from the posterior tibial vessels, the dominant proximal pedicle vessels were preserved, while the minor distal pedicle vessels were cut. The medial half of the soleus muscle was longitudinally divided along the raphe between the heads, and the distal portion was incised to elevate the proximally based hemi-split medial soleus muscle flap (Figure 3A, 3B). The elevated tibialis anterior muscle and soleus muscle flaps were sutured to cover the tibia, and a skin graft was applied over them (Figure 3C, 3D).

**Figure 4:**
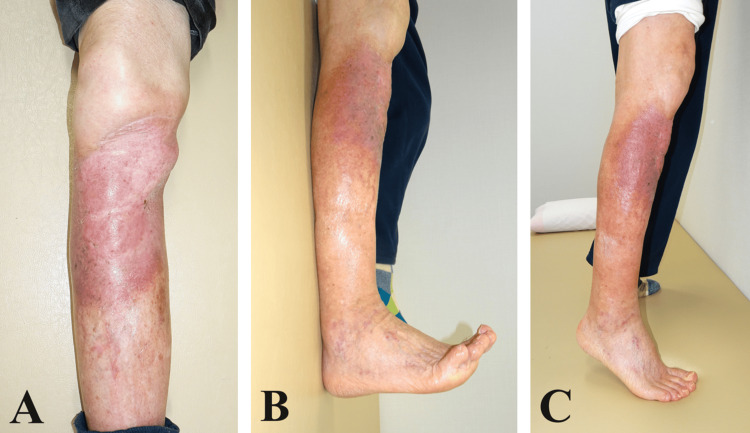
Twelve months postsurgery, (A) there was a favorable outcome without recurrence, (B) the patient had no ankle joint limitations, and (C) the patient could stand on tiptoes.

The postoperative course was uneventful, with complete survival of both the muscle flap and skin graft. Adjuvant radiotherapy was administered one month after surgery, with the patient receiving a total dose of 50 Gy. At the 12-month follow-up, the outcome was favorable, with no evidence of recurrence (Figure 4A). There was no restriction in the ankle joint range of motion (Figure 4B), and the patient demonstrated full capability to dorsiflex and plantarflex the ankle, as well as perform toe-standing (Figure 4C).

## Discussion

The reconstruction of tissue defects following STS resection in the lower leg presents a complex challenge, necessitating careful selection from a variety of techniques. Both pedicled and free flaps are employed based on defect location and extent, with comparable postoperative outcomes and complication rates reported for both methods [10,11]. A meta-analysis by Scampa et al. found no significant differences in flap necrosis rates, complication rates, aesthetic satisfaction, or postoperative infection rates between pedicled and free flaps for lower leg tissue defect reconstruction, concluding that both are reliable strategies [12]. In cases where the defect is surrounded by compromised tissue and lacks suitable recipient vessels, the cross-leg free flap offers an option for relatively extensive tissue reconstruction [13]. However, free flaps in the lower leg carry a relatively high risk of flap loss due to the scarcity of appropriate recipient vessels for anastomosis and comorbidities such as diabetes mellitus and peripheral arterial disease [14]. Free flaps may also result in donor site morbidity and are not universally available due to the specialized microsurgical expertise required. Furthermore, concerns exist regarding STS recurrence and the potential complications it poses for secondary reconstruction. Moon et al. reported a case where an anterior tibial STS was initially treated with simple excision and subsequently reconstructed using a free flap with the anterior tibial vein as a retrograde recipient vessel [15]. While the flap survived in this instance, surgeons generally hesitate to perform anastomosis in previously operated areas during reoperations. Consequently, local pedicled flaps may be preferred for lower leg reconstruction. When using local flaps, a skin island, such as a perforator flap, is desirable but only feasible for relatively small defects. Due to the limited availability of tissue for locoregional cutaneous flaps, a skin graft applied over a muscle flap proves useful for reconstructing large defects, such as those following STS resection.

The gastrocnemius muscle flap was initially selected for reconstruction based on defect location and size. However, tumor recurrence necessitated a second reconstruction. Besides the gastrocnemius, the tibialis anterior muscle and soleus muscles can potentially cover the anterior proximal third of the lower leg. The tibialis anterior muscle, supplied by anterior tibial vessels, has a type IV muscular blood supply pattern according to Mathes and Nahai's classification [16], with 8-12 similarly sized pedicles entering the muscle segmentally. As it is essential for ankle dorsiflexion, complete muscle harvest should be avoided. A partial sagittal longitudinal division allows the coverage of mid-tibial defects without compromising function [17]. The soleus muscle, supplied by perforators from posterior tibial vessels, is a type II muscle based on Mathes and Nahai's vascular supply classification [16]. Plantar flexion is weakened when the entire muscle is sacrificed, so full-width muscle flap harvest should be avoided. The hemi-soleus flap, first reported by Tobin, provides adequate soft tissue coverage while minimizing functional loss of plantar flexion [18]. The medial hemi-soleus flap, based on muscular branches of posterior tibial vessels, offers muscle flap advantages, a long arc of rotation, and an easy inset into various lower limb defects, making it a reliable option [19]. The muscle flap size (approximately 9 cm length × 5 cm width) is suitable for most small- to medium-sized defects but cannot cover large defects such as those following STS resection.

In our case, the defect was relatively large, and a single muscle flap was insufficient to cover the wound, necessitating a combination of muscle flaps. The defect was completely covered by using a hemi-split tibialis anterior muscle for the proximal portion and a hemi-split soleus muscle for the distal portion. By partially preserving each muscle, reconstruction was achieved without causing any postoperative functional disturbances in the lower extremities. As a backup reconstruction method when gastrocnemius muscle flaps cannot be utilized due to multiple prior surgeries or free flap failure, the hemi-split tibialis anterior muscle flap and hemi-split soleus muscle flap are considered useful techniques that preserve function.

## Conclusions

Reconstruction of recurrent STS in the lower leg is challenging due to the limited availability of tissue. The invasiveness of extensive tumor resection and the usage of tissue for reconstruction may cause functional impairment of the lower limbs. The hemi-split tibialis anterior and soleus muscle flaps are considered valuable methods for reconstruction, preserving lower extremity function.

## References

[1] Bridgham KM, El Abiad JM, Lu ZA (2019). Reconstructive limb-salvage surgery after lower extremity soft tissue sarcoma resection: a 20-year experience. J Surg Oncol.

[2] Chao AH, Mayerson JL, Chandawarkar R, Scharschmidt TJ (2015). Surgical management of soft tissue sarcomas: extremity sarcomas. J Surg Oncol.

[3] Uyar İ, Aksam E, Yit K (2023). Reconstruction option in complex lower extremity defects where microsurgical repair is not possible: randomized bipedicled flaps. Ulus Travma Acil Cerrahi Derg.

[4] Bibbo C (2020). The gastrocnemius flap for lower extremity reconstruction. Clin Podiatr Med Surg.

[5] Feldman JJ, Cohen BE, May JW Jr (1978). The medial gastrocnemius myocutaneous flap. Plast Reconstr Surg.

[6] Gkiatas I, Korompilia M, Kostas-Agnantis I, Tsirigkakis SE, Stavraki M, Korompilias A (2021). Gastrocnemius pedicled muscle flap for knee and upper tibia soft tissue reconstruction. A useful tool for the orthopaedic surgeon. Injury.

[7] Ezuddin NS, Pretell-Mazzini J, Yechieli RL, Kerr DA, Wilky BA, Subhawong TK (2018). Local recurrence of soft-tissue sarcoma: issues in imaging surveillance strategy. Skeletal Radiol.

[8] Fujiki M, Miyamoto S, Kobayashi E, Sakuraba M, Chuman H (2016). Early detection of local recurrence after soft tissue sarcoma resection and flap reconstruction. Int Orthop.

[9] Daigeler A, Zmarsly I, Hirsch T, Goertz O, Steinau HU, Lehnhardt M, Harati K (2014). Long-term outcome after local recurrence of soft tissue sarcoma: a retrospective analysis of factors predictive of survival in 135 patients with locally recurrent soft tissue sarcoma. Br J Cancer.

[10] Slump J, Hofer SO, Ferguson PC (2018). Flap choice does not affect complication rates or functional outcomes following extremity soft tissue sarcoma reconstruction. J Plast Reconstr Aesthet Surg.

[11] Scaglioni MF, Meroni M, Knobe M, Fritsche E (2022). Versatility of perforator flaps for lower extremity defect coverage: technical highlights and single center experience with 87 consecutive cases. Microsurgery.

[12] Scampa M, Mégevand V, Suva D, Kalbermatten DF, Oranges CM (2022). Free versus pedicled flaps for lower limb reconstruction: a meta-analysis of comparative studies. J Clin Med.

[13] Osaki T, Hasegawa Y, Tamura R (2022). Combined treatment using cross-leg free flap and the Masquelet technique: a report of two cases. Case Reports Plast Surg Hand Surg.

[14] Pu LL (2021). Free flaps in lower extremity reconstruction. Clin Plast Surg.

[15] Moon J, Lee KT, Park JW (2023). Lower leg reconstruction with free tissue transfer using reverse flow recipient vein: a case report. Int J Low Extrem Wounds.

[16] Mathes SJ, Nahai F (1981). Classification of the vascular anatomy of muscles: experimental and clinical correlation. Plast Reconstr Surg.

[17] Hallock GG (2002). Sagittal split tibialis anterior muscle flap. Ann Plast Surg.

[18] Tobin GR (1985). Hemisoleus and reversed hemisoleus flaps. Plast Reconstr Surg.

[19] Nazneen DA, Sarkar DA (2023). Exposed distal tibia coverage by reversed soleus muscle flap: our experiences. JPRAS Open.

